# Vitamin D Receptor Gene Polymorphisms in Susceptibility to Tuberculosis in the Kazakh Population in Almaty and Almaty Area

**DOI:** 10.5195/cajgh.2013.102

**Published:** 2014-01-24

**Authors:** Maxat Zhabagin, Zhannur Abilova, Ayken Askapuli, Saule Rakhimova, Ulykbek Kairov, Kulzhan Berikkhanova, Assel Terlikbayeva, Meruert Darisheva, Arike Alenova, Ainur Akilzhanova

**Affiliations:** 1 Center for Life Sciences, Nazarbayev University, Astana, Kazakhstan; 2 The Global Health Research Center of Central Asia, Almaty, Kazakhstan; 3 National Center for Tuberculosis Problems, Almaty, Kazakhstan

**Keywords:** vitamin D receptors, immune response, tuberculosis, gene polymorphism, Kazakhstan

## Abstract

**Introduction:**

Vitamin D receptor (VDR) plays an important role in activating the immune response against various infectious agents. It is known that the active metabolite of ligand receptor Vitamin D (1,25 – dihydroxyvitamin D) is encoded by VDR and helps mononuclear phagocytes to suppress the intracellular growth of *M. tuberculosis*. The VDR gene harbors approximately 200 polymorphisms, some of which are linked to differences in receptor Vitamin D uptake and therefore can be considered as candidate disease risk variants. The relation between VDR gene polymorphisms and susceptibility to TB has been studied in different populations. There is not a great deal of information regarding the association of these SNPs with TB risk in the Kazakh population. The four most commonly investigated VDR polymorphisms in association with different diseases, including susceptibility to tuberculosis, are located in exon 2 (rs2228570 or FokI), intron 8 (rs1544410 or BsmI and rs7975232 or ApaI), and exon 9 (rs731236 or TaqI). The aim of our study was to determine whether these four VDR gene single nucleotide polymorphisms were associated with TB and whether they were a risk for the development of TB in the Kazakh Population in Almaty city and Almaty area.

**Methods:**

This study was a hospital-based case-control analysis of 283 individuals (99 TB patients and 184 healthy controls). Genotyping was performed by Taqman SNP allelic discrimination using commercial TaqMan SNP Genotyping assays. Statistical analysis was conducted using SPSS Version 19.0 software.

**Results:**

Genotype frequencies for the Kazakh population are close to world (HapMap) data on Asian populations. FokI and ApaI polymorphisms genotypes tend to be associated with TB risk under the co-dominant model [OR=1.18; 95%CI: (0.68, 2.07), p=0.15] for FokI and [OR=1.33; 95%CI: (0.61, 2.91), p=0.6] for ApaI. No significant association between the disease and TaqI, BsmI genotypes was observed.

**Conclusions:**

In summary, we explored potential associations between SNPs in the VDR (FokI, ApaI) gene and susceptibility to tuberculosis in the Kazakh Population, which requires further detailed analysis with a larger sample size and greater geographic diversity including other regions of Kazakhstan.

